# Mixture of Experts with Entropic Regularization for Data Classification

**DOI:** 10.3390/e21020190

**Published:** 2019-02-18

**Authors:** Billy Peralta, Ariel Saavedra, Luis Caro, Alvaro Soto

**Affiliations:** 1 Department of Engineering Science, Andres Bello University, Santiago 7500971, Chile; 2 Department of Engineering Informatics, Catholic University of Temuco, Temuco 4781312, Chile; 3 Department of Computer Sciences, Pontifical Catholic University of Chile, Santiago 7820436, Chile

**Keywords:** mixture-of-experts, regularization, entropy, classification

## Abstract

Today, there is growing interest in the automatic classification of a variety of tasks, such as weather forecasting, product recommendations, intrusion detection, and people recognition. “Mixture-of-experts” is a well-known classification technique; it is a probabilistic model consisting of local expert classifiers weighted by a gate network that is typically based on softmax functions, combined with learnable complex patterns in data. In this scheme, one data point is influenced by only one expert; as a result, the training process can be misguided in real datasets for which complex data need to be explained by multiple experts. In this work, we propose a variant of the regular mixture-of-experts model. In the proposed model, the cost classification is penalized by the Shannon entropy of the gating network in order to avoid a “winner-takes-all” output for the gating network. Experiments show the advantage of our approach using several real datasets, with improvements in mean accuracy of 3–6% in some datasets. In future work, we plan to embed feature selection into this model.

## 1. Introduction

Machine learning, one of the fastest growing areas in computer science, refers to the study of computing methods for the recognition of patterns in data, as well as algorithms that allow machines to perform computing tasks autonomously [1]. Machine learning methods have been extended to various application domains, such as microbiology [2], web mining [3], spam detection [4], and recommendation systems [5]. An important task in this discipline is automatic data classification, which consists of learning a model that associates input data with a set of labels. There are multiple techniques for automatic data classification, such as neural networks, support vector machines, and ensemble-based models.

Mixture-of-experts (MoE) is an ensemble-based classification technique proposed by [6]. MoE is a probabilistic model composed of a set of networks that stratifies the input space and assigns a local classifier to each partition, leading to a “divide-and-conquer” strategy. MoE has been used in multiple applications, such as text recognition [7], time series prediction [8], and speech pathology recognition [9]. In [10], a set of intermediate MoEs led to a significant increase in the capacity parameter of deep models without a critical increase in computing capacity. This work uses a sparse mixture-of-experts for distributing multiple deep neural networks efficiently.

**Figure 1:**
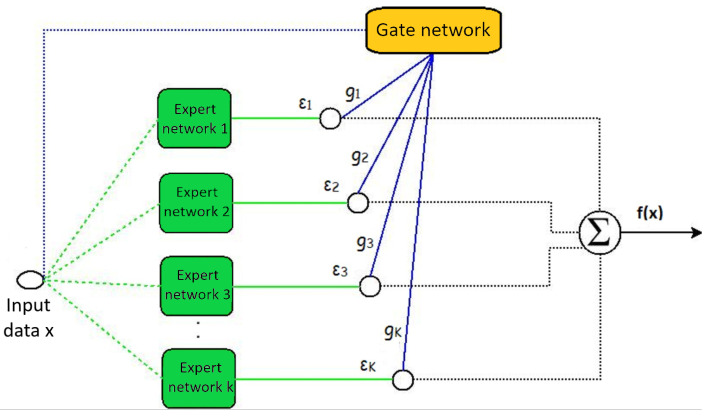
Mixture-of-experts (MoE) architecture.

Mixture-of-experts models have two basic components: expert and gate networks, which can be visualized in Figure 1. The green squares represent the expert networks with the parameter $\varepsilon_{i}$, whose function is to learn to predict the class of the input data. On the other hand, the gate network with parameter $g_{i}$, symbolized by the yellow rectangle, assigns weights to each of the experts and thus generates the resulting classification.

The MoE model learns a probabilistic ensemble of classifiers, each of which is learned using partitioned input data. The result is weighted according to the gate network outputs; a weight is interpreted as the importance of the expert for the classification of an item of input data. Ensemble models have two possible environments: competitive and cooperative [11]. In competitive environments, there is a tendency for only one or very few ensemble components to be important; in cooperative environments, on the other hand, it is expected that many components will be important. Due to the typical use of the softmax function in the gate network, MoE generates a competitive environment in which, typically, the outputs are vectors, with few nonzero outputs [12].

This work proposes a variant of the classical mixture-of-experts. In the proposed model, the gating network entropy is maximized during the parameter training process; therefore, the typical competitive environment becomes more cooperative. Our idea is to favor overlapping local experts for each input datum in order to capture more complex data patterns. In particular, we incorporated the use of Shannon entropy, as it can represent the degree of complexity of a probability function. Entropic regularization consists of maximizing the entropy measure enforced by the gate network output to avoid concentrating on a single expert, which is the behavior of the classical MoE based on softmax functions. Another alternative is given by Renyi entropy, which is a generalization of Shannon entropy, and it leads to a more flexible model. However, Renyi entropy requires an extra parameter (α) that is not easily estimated for each dataset. For this reason, we expect that Renyi entropy will be explored in a future work.

The idea of including regularizing entropy in conjunction with the optimization of the cost function to improve the performance of a learning task was previously explored in the context of latent probabilistic semantic analysis [13] by minimizing the entropy of the weighting of a mixture of latent factors in order to increase weighting sparsity. Studies have also been performed in the context of semi-supervised learning [14,15]; these studies minimized the entropy of the conditional probability of the classes on the basis of the data to minimize the class uncertainty of the unsupervised data. However, in contrast to related works which focused on entropy minimization, no works have been found that added regularization by entropy maximization to the mixture-of-experts model, as we propose here.

## 2. Mixture-of-Experts

The mixture-of-experts model is composed of a set of expert networks, each of which solves a part of the problem using an approximation function in the input space. The main idea of MoE is to obtain local models, each specialized in a particular data region. The model assumes *N* labeled training examples, where the *n*th datum is given by the tuple $\left(x_{n},y_{n}\right)$ such that $x_{n}\in R^{D}$ and $y_{n}\in C$, which is equal to the set of class labels with cardinality *Q* and consists of $\left\{c_{1},c_{2}\ldots c_{Q}\right\}$. Assuming that the *i*th expert is represented by $m_{i}$, where $i\in \left\{1,2,\ldots ,K\right\}$ and *K* is the number of experts, we establish a probabilistic expression that models the output *y*, given the expert $m_{i}$ and the data input *x*, as follows:

(1) $$\begin{array}{c} p\left(y|x,m_{i}\right)=p\left(y=c_{l}|x,m_{i}\right),i=1,2,\ldots ,K \end{array}$$

where $c_{l}$ corresponds to the *l*th class. Following Moerland [16], as we apply MoE to classification modeling, we use a multinomial density function. Therefore, the function of the expert network is defined asx

(2) $$\begin{array}{c} p\left(y=c_{l}|x,m_{i}\right)=\frac{exp(\omega_{li}^{T}x)}{\sum_{j=1}^{Q}exp\left(\omega_{ji}^{T}x\right)} \end{array}$$

Specifically, $\omega_{li}$ represents the parameter vector of the expert network that depends on class $c_{l}$ and expert *i*. Analogous to the expert network, the function of the gate network represents the conditional probability of datum *x* given by expert $m_{i}$. This probability is represented by function $g_{i}$ and formulated as follows:

(3) $$\begin{array}{c} p\left(m_{i}|x\right)=g_{i}\left(x,\nu \right)=\frac{exp(\nu_{i}^{T}x)}{\sum_{j=1}^{K}exp\left(\nu_{j}^{T}x\right)} \end{array}$$

The variable $\nu_{i}$ is the parameter vector of the gate components, where $i\in \left\{1,2,\ldots ,K\right\}$. The final output of the network corresponds to the sum of the outputs of the experts that are weighted by the gate network. Considering a probabilistic interpretation [17], the conditional probability of output *y* given data input *x* is calculated as follows:

(4) $$\begin{array}{ccc} p(y|x) & = & \sum_{i=1}^{K}g_{i}\left(x,\nu \right)p\left(y|x,\omega_{i}\right) \end{array}$$

The parameters of the gate network, $\nu_{i}$, and the expert network, $\omega_{i}$, are typically estimated using the expectation–maximization (EM) algorithm.

### EM Algorithm for Mixture-of-Experts

First, we need to maximize the expected log-likelihood function of the training data considering the conditional probability p(y|x) [18] as given by

(5) $$\begin{array}{c} \hat{\ell }\left(\nu ,\omega \right)=ln\left(L\left(\nu ,\omega \right)\right)=\sum_{n=1}^{N}ln\sum_{i=1}^{K}g_{i}\left(x_{n},\nu \right)p\left(y_{n}|x_{n},\omega_{i}\right) \end{array}$$

We assume that the assignment of data to experts is known by means of the hidden variable *z*, with $z_{ni}$ equal to one if the *n*th data point is generated by expert *i*th expert; otherwise, $z_{ni}$ is equal to zero. Then, the complete log-likelihood function is formulated as

(6) $$\begin{array}{c} \hat{\ell }\left(\nu ,\omega |x,z\right)=\sum_{n=1}^{N}\sum_{i=1}^{K}z_{ni}\;ln\left(g_{i}\left(x_{n},\nu \right)p\left(y_{n}|x_{n},\omega_{i}\right)\right) \end{array}$$

The steps of the EM algorithm for MoE are defined as follows: **E Step:** The expected value of the assignment variable $z_{ni}$ is inferred by applying the Bayes theorem:

(7) $$\begin{array}{ccc} E(z_{i}^{n}) & = & \frac{p\left(y_{n}|z_{ni}=1,x_{n}\right)p\left(z_{ni}=1|x_{n}\right)}{p(y_{n},x_{n})} \end{array}$$

Replacing the probabilities of the numerator of Equation (7), we establish that $p(y_{n}|z_{ni}=1,x_{n})$ is equivalent to the probability density function of expert *i*, $p(y_{n}|x_{n},\omega_{i})$, and $p(z_{ni}=1|x_{n})$ is equivalent to the output of the gate network $g_{i}\left(x_{n},\nu \right)$. Finally, the probability $p(z_{ni}=1|y_{n},x_{n})$ is defined as the a posteriori probability of the *i*th expert given the instance–label pair ($x_{n},y_{n}$) and is represented by the variable $\pi_{ni}$ known as responsibility.

**M Step:** The expected complete log-likelihood function for training data is defined as:

(8) $$\begin{array}{c} E=\sum_{n=1}^{N}\sum_{i=1}^{K}\pi_{ni}\left[ln\left(g_{i}\left(x_{n},\nu \right)\right)+ln\left(p\left(y_{n}|x_{n},\omega_{i}\right)\right)\right] \end{array}$$

After applying calculus, the cost function derivative with respect to the gate network parameters is

(9) $$\begin{array}{ccc} \frac{\partial E}{\partial \nu_{i}} & = & \sum_{n=1}^{N}\sum_{i=1}^{K}\pi_{ni}\frac{g_{i}{\left(x_{n},\nu \right)}^{{}^{\prime }}}{g_{i}\left(x_{n},\nu \right)} \end{array}$$

Similarly, the cost function derivative with respect to the expert network parameters is given by

(10) $$\begin{array}{ccc} \frac{\partial E}{\partial \omega_{ji}} & = & \sum_{n=1}^{N}\sum_{i=1}^{K}\pi_{ni}\;\frac{p\left(y_{n}|x_{n},\omega_{i}\right){)}^{{}^{\prime }}}{p(y_{n}|x_{n},\omega_{i})} \end{array}$$

To find the model parameters, we equate both of the previous derivatives to zero. Using the weighted least squares method and approximating the softmax function for the gate network parameters [16], we have:

(11) $$\begin{array}{ccc} W_{j}^{T} & = & {\left(X^{T}\Pi_{j}X\right)}^{-1}X^{T}\Pi_{j}\;ln\left(Y\right) \end{array}$$

(12) $$\begin{array}{ccc} V^{T} & = & {\left(X^{T}X\right)}^{1}X^{T}ln\left(\Pi_{j}\right) \end{array}$$

where *X* represents the input data with the dimension N×D; $W_{j}$ is the parameter matrix of the *j*th expert with the size Q×I; and *Y* is the class matrix with the dimension N×Q. Finally, $\Pi_{j}$ is the responsibilities matrix with the size K×D.

## 3. Mixture-of-Experts with Entropic Regularization

The consequence of using the softmax function for the gate network of the mixture-of-experts model is that each individual datum is generated by a single expert. The reason is that the softmax function generates a “*winner-take-all*” behavior [19] among the gate outputs. Nonetheless, we think that the data patterns can be complex enough that each data input can be better modeled by a set of experts than by just one expert. Therefore, we propose favoring this superposition of experts by controlling the entropy of the gate network output. In particular, we propose the minimization of the cost classification and the simultaneous maximization of the entropy of the gate network outputs.

Our rationale is that a gate network output given by a vector with only one active gate (i.e., a vector filled with zeros excepting one component, which is equal to one) has a lower entropy than a gate function output where there are many active gates (i.e., a vector where few components are equal to zero). Therefore, we maximize the entropy of the gate network output in order to avoid outputs with very few active gates. On the other hand, the minimization of the gate network error forces MoE to specialize in a few gate functions. By combining the two terms—the gate network cost based on the softmax function and the entropy of the gate network outputs—we obtain an intermediate solution given by a superposition of local expert functions. We call this the mixture-of-experts with entropic regularization or “entropic mixture-of-experts” (EMoE). We develop this approach below.

Considering the cost equation associated with the gate network, to which we add the Shannon entropy with the weight $\hat{\lambda }$, which represents the entropy degree of a continuous variable, we obtain:

(13) $$\begin{array}{ccc} E_{g} & = & \sum_{n=1}^{N}\sum_{i=1}^{K}\pi_{ni}\;ln\left(g_{i}\left(x_{n},\nu \right)\right)+\hat{\lambda }\sum_{n=1}^{N}\sum_{i=1}^{K}g_{i}\left(x_{n},\nu \right)log_{2}\left(g_{i}\left(x_{n},\nu \right)\right)\\ & = & \sum_{n=1}^{N}\sum_{i=1}^{K}\pi_{ni}\;ln\left(g_{i}\left(x_{n},\nu \right)\right)+\lambda \sum_{n=1}^{N}\sum_{i=1}^{K}g_{i}\left(x_{n},\nu \right)ln\left(g_{i}\left(x_{n},\nu \right)\right) \end{array}$$

In the expression $log_{2}\left(g_{i}\left(x_{n},\nu \right)\right)=ln\left(g_{i}\left(x_{n},\nu \right)\right)/ln\left(2\right)$, the constants ln(2) and $\hat{\lambda }$ are absorbed by λ, which corresponds to the entropic regularization constant. For efficiency reasons, we differentiate the components of softmax and entropy functions in $E_{g}$:

(14) $$\begin{array}{c} \underset{\alpha }{\underset{︸}{\sum_{n=1}^{N}\sum_{i=1}^{K}\pi_{ni}ln\left(g_{i}\left(x_{n},\nu \right)\right)}}+\underset{\beta }{\underset{︸}{\sum_{n=1}^{N}\sum_{i=1}^{K}\lambda \;g_{i}\left(x_{n},\nu \right)ln\left(g_{i}\left(x_{n},\nu \right)\right)}} \end{array}$$

The first term of Equation (14), α, corresponds to the cost associated with the adjustment of the parameters of the gate network. The second term, β, corresponds to the entropic regularization added to the model. Now, we proceed to freeze a dependent term of $g_{i}$ from the previous iteration t−1 to form the following:

(15) $$\begin{array}{c} \beta =\sum_{n=1}^{N}\sum_{i=1}^{K}\lambda \;g_{i}^{t-1}\left(x_{n},\nu \right)ln\left(g_{i}\left(x_{n},\nu \right)\right) \end{array}$$

By replacing the version of β of the previous iteration in Equation (15), we obtain:

(16) $$\begin{array}{ccc} E_{g} & = & \underset{\alpha }{\underset{︸}{\sum_{n=1}^{N}\sum_{i=1}^{K}\pi_{ni}ln\left(g_{i}\left(x_{n},\nu \right)\right)}}+\\ & & \underset{\beta }{\underset{︸}{\sum_{n=1}^{N}\sum_{i=1}^{K}\lambda \;\overset{t-1}{\underset{i}{g}}\left(x_{n},\nu \right)ln\left(g_{i}\left(x_{n},\nu \right)\right)}} \end{array}$$

Considering that the gate network has a linear dependence of $s_{i}=\nu_{i}^{T}x$, we derive $E_{g}$ with respect to $\nu_{i}^{T}$, giving

(17) $$\begin{array}{c} \sum_{n}\left(\pi_{ni}-g_{i}\left(x_{n},\nu \right)\right)x_{n}\;+\lambda \sum_{n}\left(g_{i}\left(x_{n},\nu \right)-g_{i}^{t-1}\left(x_{n},\nu \right)\right)x_{n}=0 \end{array}$$

Since Equation (17) cannot be directly solved, we apply the same solution as that of the classical MoE given by the replacement of the outputs by the outputs’ logarithm [16] to obtain

(18) $$\begin{array}{c} \sum_{n}\left(ln\left(\pi_{ni}\right)-s_{ni}\right)x_{n}\;+\lambda \sum_{n}\left(s_{in}-s_{ni}^{t-1}\right)x_{n}=0 \end{array}$$

Therefore, the change in the M step for the proposed variant of mixture-of-experts is given in matrix terms by

(19) $$\begin{array}{c} V=\frac{1}{1-\lambda }{\left(X^{\prime }X\right)}^{-1}X^{\prime }ln\left(\Pi \right)-\frac{\lambda }{1-\lambda }V^{t-1} \end{array}$$

where $V^{t-1}$ is the optimal parameter of the gate network in the previous iteration t−1. The addition of the Shannon entropy term can generate a non-convex cost function, since its weight (λ) can have a negative sign; this generates a difference in convex functions when the entropic cost function is added. This weight can be negative because it is selected considering the obtained accuracy in a validation dataset. As we observe in some experiments, it is possible that a regular MoE can produce dense gating network outputs. Therefore, the best option is to decrease the entropy rather than increasing it, leading to a negative weight. As a consequence, parameter training may become more unstable. Nonetheless, we typically observe in the experiments that the proposed model obtains denser gating networks outputs; moreover, in several cases, the classification accuracy of the EMoE model exceeds the typical model given by MoE.

## 4. Experiments

This section details the experiments that were implemented to test the classification accuracy of the proposed technique, EMoE, in comparison with the traditional MoE. We also analyze the behavior of its likelihood function and the Shannon entropy of the gate network outputs. The experiments were performed using real datasets (mainly from the UCI repository) with diverse characteristics, such as the number of records and variables.

The classification results were obtained by performances using two-level nested stratified cross-validation with 30 external folds and 10 internal folds. In particular, in the case of EMoE, we used the internal 10-fold cross-validation to pick the entropy regularization constant that has the values $\left\{\pm 2^{-7},\pm 2^{-6},\pm 2^{-5},\pm 2^{-4},\pm 2^{-3},\pm 2^{-2},\pm 2^{-1},2^{0}\right\}$. After the entropy regularization constant was selected, we used the complete training set to estimate parameters for the expert and gate functions, which were evaluated using test sets following the external 30-fold cross-validation scheme.

In particular, we compared the classic mixture-of-experts (MoE) and the entropic mixture of experts (EMoE) techniques for the following aspects:

- Log-likelihood: we measured the value of the log-likelihood function for each iteration with both methods. Specifically, we analyzed the convergence of the algorithms.
- Average accuracy: we measured the accuracy of the prediction by examining the average of the results given by the cross-validation procedure. We analyzed these values considering the number of experts, which corresponds to 10, 20, 30, 40, and 50 experts.
- Average entropy: we measured the average entropy value of the gate network outputs. We used these values to visually analyze the entropy behavior when it is incorporated into the cost function.

Furthermore, we show the optimal parameters for all datasets and summarize the accuracy results for the best parameters with different numbers of experts. The datasets used in these experiments are detailed below.

### 4.1. Datasets

The datasets were mostly extracted from the UCI machine learning repository [20]. They have different dimensionalities in order to explore the behavior of the proposed method for diverse conditions. Table 1 shows the details of these datasets.

### 4.2. Log-Likelihood Analysis

**Figure 2:**
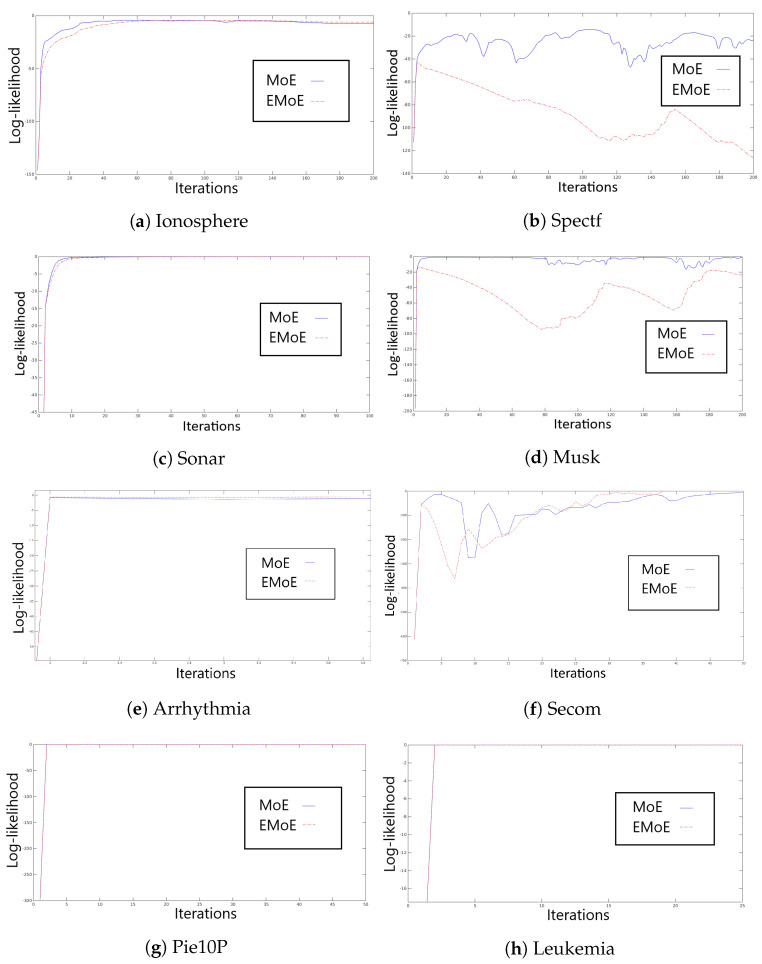
Log-likelihood values with 20 experts for the classical MoE and the entropic MoE (EMoE) for all datasets. In these experiments, we mainly used 50 iterations.

In this subsection, we assess the log-likelihood behavior observed during the execution of the EM algorithm for MoE and EMoE. Figure 2 shows the log-likelihood of the data during the training process for all databases with 20 experts and at least 50 iterations.

The log-likelihood analysis indicates that the Ionosphere dataset reaches convergence in a few iterations due to its low dimensionality. We observe that the MoE log-likelihood is higher than the EMoE log-likelihood for every iteration until iteration 100. In the case of Spectf, MoE shows slightly irregular behavior; however, EMoE is even more irregular. The Sonar dataset shows an increase in the log-likelihood function for both MoE and EMoE for up to 30 iterations. The Musk dataset presents an incremental log-likelihood for up to 50 iterations for MoE, while EMoE has irregular behavior. In the Arrhythmia dataset, the convergence of the EM algorithm for both methods is found in several iterations. Secom shows an irregular convergence for both methods; after 50 iterations, the log-likelihood becomes more regular. In the cases of PIE10P and Leukemia, convergence is reached in less than 10 iterations. In general, we observe that the log-likelihood evolution is variable, where the addition of an entropic penalty tends to make the EM algorithm less stable in the EMoE than in the MoE. In all subsequent experiments, 50 iterations were used to train the models, except in the last two datasets, for which 5 iterations were used.

### 4.3. Accuracy Analysis

In this subsection, we first detail the search for the entropy regularization constant λ for EMoE. This process was performed in each internal layer of the cross-validation procedure, using only the training set in each external fold. The results of this search with the respective mode of the constant values for different numbers of experts are shown in Table 2. The entropy penalty is found inside a grid with values following an exponential rate from $2^{-7}$ to $2^{7}$ using powers of 2.

In summary, we observe that the best values of the entropy regularization constant λ vary according to the dataset. We observe negative values, such as −128 for 20 experts with Ionosphere; high values, such as 128 for 10 experts with Pie10P; and low values, such as 0.5 for 10 experts with Arrhythmia. The values obtained were used in the following experiment. Interestingly, we find that the optimal values of λ correspond to both negative and positive values. We think that this is because the weight of Shannon entropy can have valid positive values that computed to be negative according to the validation procedure. In general, we find that the experimental entropy of the gate network outputs for all datasets is bigger for the EMoE model than the MoE model.

Finally, Table 3 shows the classification accuracy using the two-level nested cross-validation procedure. We observe that the proposed entropic mixture-of-experts improves the results of the classical mixture-of-experts in almost all cases by approximately 1–4%. In the Ionosphere dataset, the performance of EMoE is higher than that of MoE in all cases, with the best performance reached with 30 experts. MoE reaches the optimum with 20 experts, with a difference of 3% in favor of EMoE. In the case of Spectf, the difference is more variable, but EMoE again performs better than MoE for all configurations: both EMoE and MoE reach the best performance with 20 experts, with a difference of 6% in favor of EMoE. In the Sonar dataset, the accuracies are similar for both algorithms; the best performance for both algorithms is given by 40 experts. In the case of Musk, EMoE is superior to MoE in most cases: EMoE reaches its best accuracy with 30 experts and MoE with 50, with similar values. In the Arrhythmia dataset, although the entropies behaved similarly, we observe that EMoE improves upon MoE in all cases; the best configuration of EMoE is given by 50 experts, while MoE reaches its best with 20, with a 6% difference in favor of EMoE. This behavior is repeated in Secom, where the entropies are again similar in their behaviors; when all configurations are considered, EMoE again outperforms MoE. Both present their greatest accuracy with 50 experts, with a difference of 3% in favor of EMoE. In the cases of Pie10P and Leukemia, the performance remains similar for both algorithms.

In this experiment, EMoE typically outperforms MoE. This confirms our intuition about the relevance of overlapping experts for improving accuracy. In general, we note that in the datasets with the highest dimensionality, EMoE does not achieve any improvement. We hypothesize that a high dimensionality causes a greater possibility of overfitting, which affects our proposed model since it assumes the use of more complex models for model input data. This suggests that the use of embedded variable selection in entropic mixture-of-experts could improve the presented results.

### 4.4. Visual Analysis of Average Entropy of Gate Network Outputs

**Figure 3:**
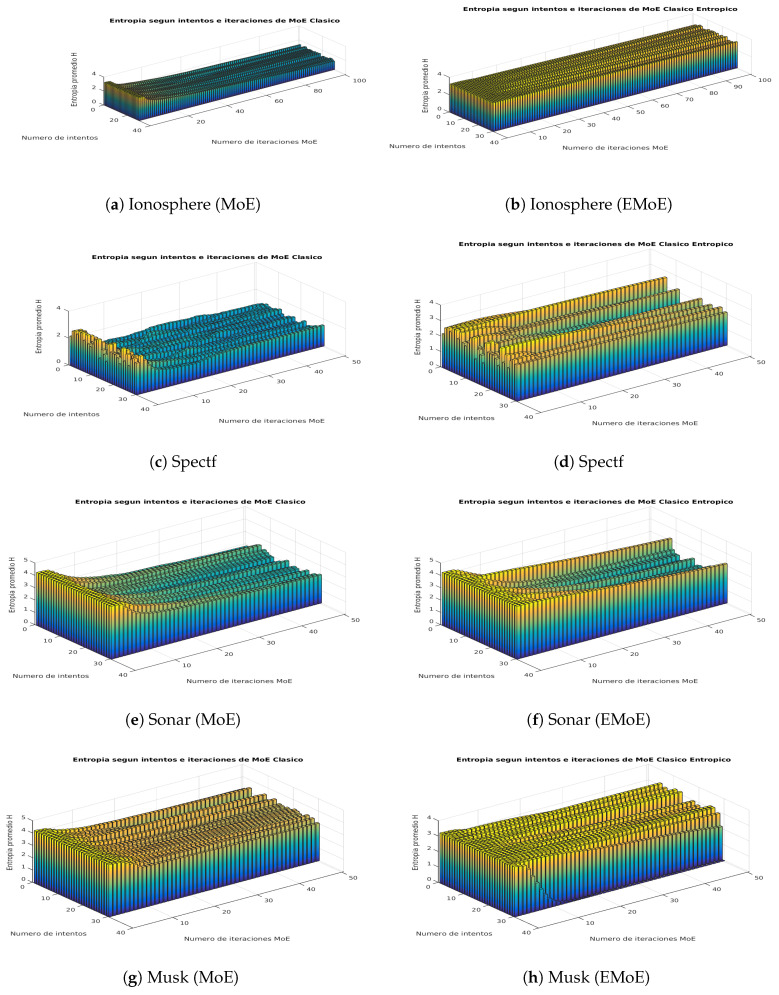
Average entropy scores in the network gate outputs for the Ionosphere, Spectf, Sonar, and Musk datasets in the MoE and EMoE models with 10 experts.

**Figure 4:**
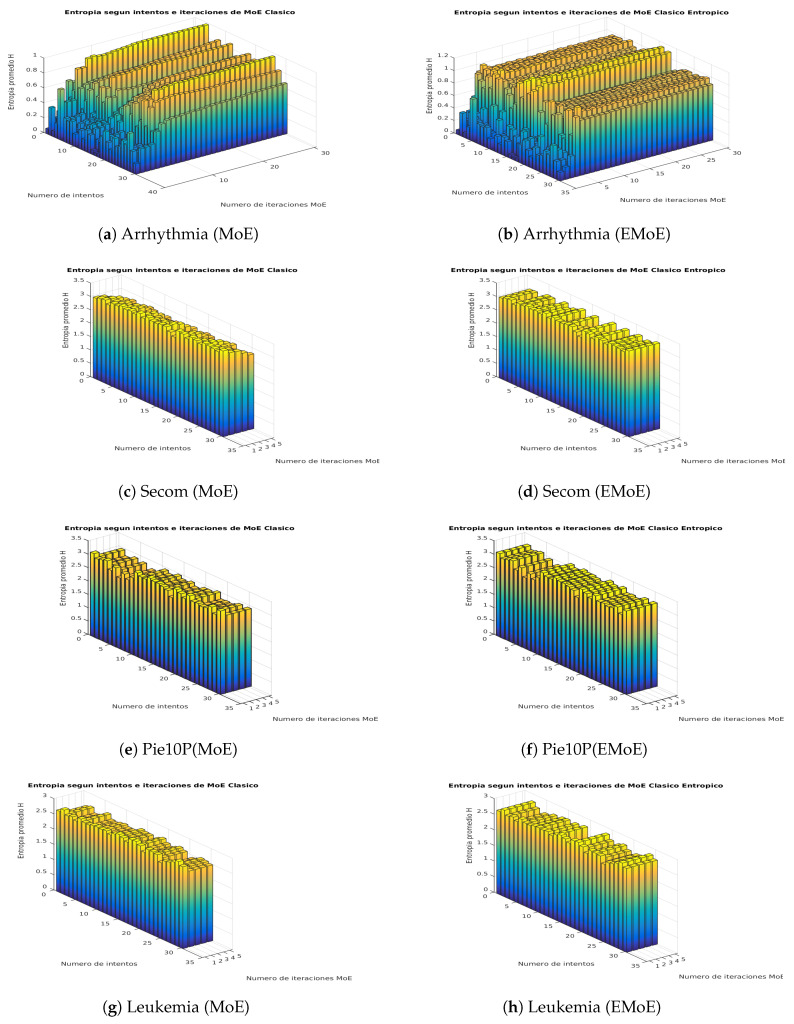
Average entropy scores in the network gate outputs for the Arrhythmia, Secom, Pie10P, and Leukemia datasets in the MoE and EMoE models with 10 experts.

This subsection shows visually how the entropy in the gate network outputs is affected by the proposed formulation. We propose a score on that is based on the average Shannon entropy of the gate network. This score is calculated for each iteration *i* of the EM algorithm, in which Shannon entropy is calculated for the gate network outputs for each data, and then these values are averaged. Figure 3 and Figure 4 present the results for both algorithms for the iterations of the algorithms and the different partitions according to the cross-validation procedure.

The plot of average entropy scores for the Ionosphere dataset shows that while the entropy decreases in MoE, it remains constant in EMoE. In Spectf, the previous trend remains, although with greater variability. In Sonar, the trend becomes even more variable, and, in many cases, there is a similar entropy pattern for both techniques. In Musk, the entropy generally decreases in MoE, while, in EMoE, an increasing trend is observed in some cases. In Arrhythmia, the trend is increasing in both cases. In Secom, the entropy in MoE tends to decrease, while in EMoE it tends to remain the same. In Pie10P, as in Leukemia, the average entropy tends to be similar for both techniques. In general, we observe that in high-dimensionality datasets, the entropy maintains similar patterns for both algorithms, while in databases with smaller dimensionalities, entropy tends to be more uniform for EMoE than for MoE.

## 5. Conclusions

This paper proposes EMoE, a regularized variant of mixture-of-experts in which entropy penalization is applied to gate network outputs using Shannon entropy in order to obtain more overlapping experts. Our experiments provide evidence that the EMoE technique improves on the classification accuracy of the classical mixture-of-experts. The results for a diverse set of real datasets indicate a greater average accuracy. In this respect, the proposed technique demonstrates greater utility when the datasets do not have a large number of dimensions. In datasets with high dimensionality, there is no significant difference in comparison with classical MoE models. We observe that the optimal value of the regularization constant λ causes an increase in the average entropy of the gate function outputs compared with the classical MoE scheme. The experiments also show that there is a tendency toward negative values of the regularization constant in datasets of small dimensionality (Ionosphere and Musk) and mostly positive values in datasets of higher dimensionality (Arrhythmia, Leukemia, and Pie10P). As future work, we plan to implement the proposed penalty in mixtures of experts with an embedded selection of variables. Another avenue of future research is exploration of the use of more expressive entropy expressions, such as Renyi entropy.

## Figures and Tables

**Table 1:** Real datasets used in experiments.

Dataset Name	Number of Instances	Dimensionality	Number of Classes
Ionosphere	351	33	2
Spectf	267	44	2
Sonar	208	61	2
Musk-1	486	168	2
Arrhythmia	452	279	16
Secom	1567	471	2
PIE10P	210	1000	10
Leukemia	75	1500	2

**Table 2:** Summary of the best parameters found by the grid search procedure for each of the datasets analyzed and the number of experts.

Dataset	K = 10	K = 20	K = 30	K = 40	K = 50
Ionosphere	−32	−128	−16	−32	−128
Spectf	128	128	−2	−1.5	8
Sonar	−1	−1	64	−1.5	−2
Musk	32	−32	−32	−16	−16
Arrhythmia	0.5	0.5	0.5	0.5	0.5
Secom	8	4	8	8	32
PIE10P	128	128	128	128	128
Leukemia	8	128	64	32	128

**Table 3:** Average classification accuracy (plus its standard deviation), using 30-fold stratified cross-validation for the classical MoE and EMoE. The accuracies are obtained considering a different number of experts *K* (K=10,20,30,40,50). The best result per dataset and number of experts is shown in bold.

Dataset	K = 10		K = 20		K = 30		K = 40		K = 50	
	MoE	EMoE	MoE	EMoE	MoE	EMoE	MoE	EMoE	MoE	EMoE
Ionosphere	85.1% (0.022)	**88.4**% (0.015)	87.9% (0.025)	**90.1**% (0.023)	86.9% (0.024)	**91.0**% (0.025)	87.3% (0.020)	**90.7**% (0.023)	87.6% (0.029)	**91.1**% (0.026)
Spectf	70.6% (0.067)	**72.8**% (0.073)	72.7% (0.044)	**78.0**% (0.127)	68.0% (0.067)	**73.2**% (0.155)	71.0% (0.086)	**75.5**% (0.075)	72.5% (0.082)	**74.8**% (0.093)
Sonar	**67.5**% (0.046)	**67.5**% (0.040)	67.2% (0.038)	**67.6**% (0.047)	**69.2**% (0.043)	69.0% (0.041)	69.24% (0.052)	**69.28**% (0.047)	67.5% (0.059)	**67.9**% (0.059)
Musk	75.7% (0.031)	**75.8**% (0.030)	75.9% (0.024)	**76.1**% (0.027)	75.8% (0.022)	**76.1**% (0.017)	76.6% (0.033)	**76.7**% (0.037)	**77.4**% (0.034)	77.2% (0.032)
Arrhythmia	48.2% (0.035)	**49.7**% (0.033)	51.3% (0.048)	**55.1**% (0.063)	48.3% (0.032)	**56.5**% (0.058)	49.8% (0.028)	**55.0**% (0.063)	50.3% (0.035)	**57.0**% (0.038)
Secom	88.8% (0.012)	**92.1**% (0.008)	89.1% (0.010)	**92.2**% (0.010)	89.2% (0.014)	**92.3**% (0.009)	89.0% (0.012)	**92.4**% (0.009)	89.6% (0.012)	**92.7**% (0.010)
PIE10P	**100**% (0)	**100**% (0)	**99.96**% (0.001)	**99.96**% (0.001)	**100**% (0)	**100**% (0)	**100**% (0)	**100**% (0)	**100**% (0)	**100**% (0)
Leukemia	**80.8**% (0)	**80.8**% (0)	**80.6**% (0.001)	80.5% (0.001)	**98.2**% (0)	**98.2**% (0)	**97.4**% (0)	**97.4**% (0)	**98.3**% (0)	**98.3**% (0)
